# Safety evaluation of rail transit vehicle system based on improved AHP-GA

**DOI:** 10.1371/journal.pone.0273418

**Published:** 2022-08-24

**Authors:** Sihui Dong, Fei Yu, Kang Wang

**Affiliations:** 1 School of Traffic and Transportation Engineering, Dalian Jiaotong University, Dalian, China; 2 College of Safety Science and Engineering, Xi’an University of Science and Technology, Xi’an, China; Tsinghua University, CHINA

## Abstract

The rail transit vehicle system is an important subsystem with the most frequent operation accidents and the most direct impact on passengers. Based on the particularity of the vehicle system and the complexity of the system, the hierarchical analysis method (AHP) is used to evaluate its safety. High-order judgment matrix often has inconsistency, and the judgment matrix consistency guarantee is the key to the hierarchical analysis method applied. Based on the hierarchical analysis principle, this paper corrects the inconsistency judgment matrix and realizes the optimization calculation based on the genetic algorithm. This paper constructs a vehicle system safety evaluation index system including 26 indexes at three layers and uses the fuzzy comprehensive evaluation method to evaluate the system safety level. The results show that the calculation results based on the improved AHP-GA are significantly better than that based on the conventional AHP method. The comprehensive evaluation conclusion of the case is "average", and the safety level of the vehicle system of the case enterprise needs to be strengthened.

## Introduction

**Fig 1:**
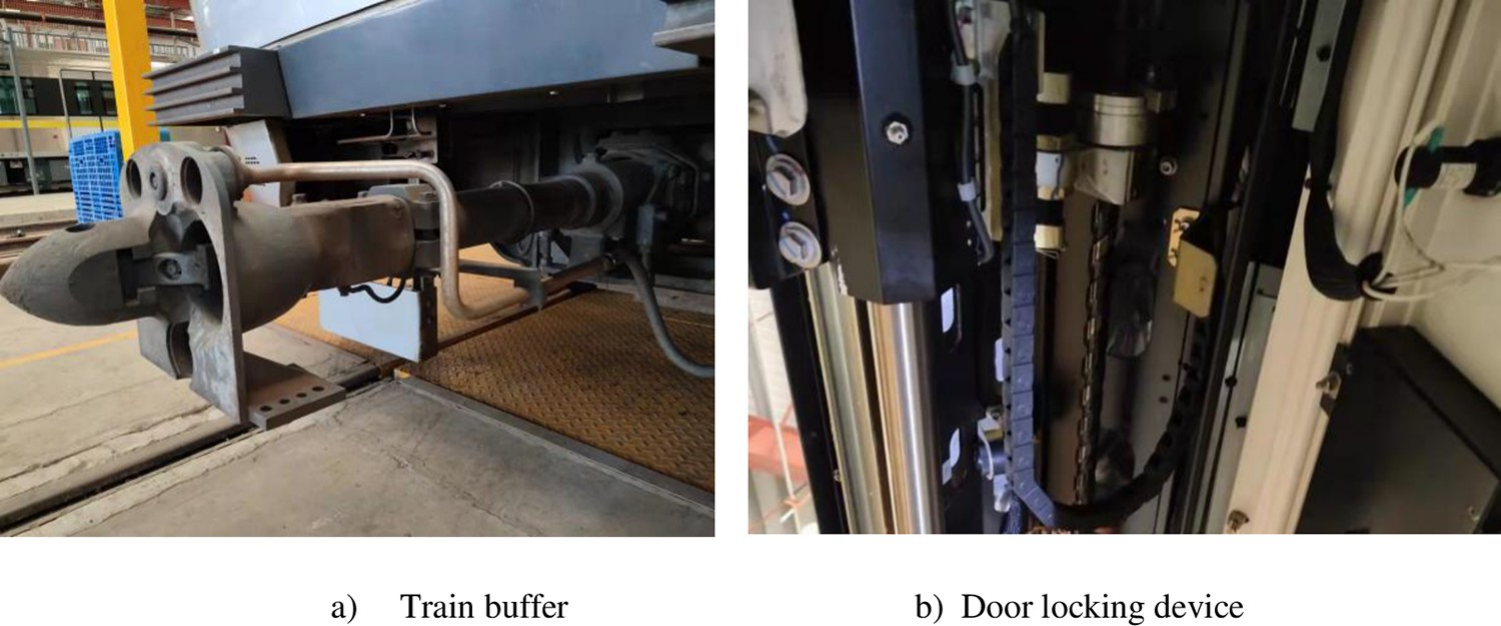
Photo of the Dalian metro scene. a) Train buffer, b) Door locking device.

Rail transit is the vehicle with the strongest carrying capacity in the public transport mode and has the characteristics of high efficiency and energy saving [1]. With the gradual development of rail transit industry, the use of rail transit vehicles is gradually expanding [2]. Vehicle system equipment plays an important role in vehicle operation [3]. The vehicle system is an integrated system including electrical system, traction system, connection device, braking system, signal system, safety protection system, etc., as shown in Fig 1. If safety risks occur in any part, the whole system may become invalid, even result in accidents.

Pang puts forward that signal failure, vehicle failure, passenger reasons and other aspects are the main manifestations of domestic subway operation accidents or failures, accounting for 74.2% of the total [4]. Wang believes that rail transit equipment failure is one of the important factors in subway operation accidents [5]. After analyzing the causes of rail traffic accidents, scholars have analyzed the equipment of the vehicle system in order to conduct further research on the rail transit vehicle system. V Nezevak conducted simulation modeling analysis on the interaction between the electric locomotive and electric traction system [6]. Traction drive system is one of the core systems of vehicle. Lin studied the reliability of vehicle traction systems [7]. The active braking control system of high-speed trains is essential to ensure safety, Chen proposed an adaptive slip rate estimation method for active braking control based on improved extended state observer [8]. Wang studied the problem of high-speed train emergency braking control based on wireless sensor network and established an emergency braking control model for analysis [9]. Alireza analyzed the data acquisition techniques used to detect the condition of rail transit wheels [10]. Railway axles are vital parts of passenger or freight railway car, Zoran analyzed the causes of railway axle failure [11]. Tang used HyperMesh and OptiStruct and other finite element software to establish the finite element model of the container of the online cleaning machine of rail transit. They analyzed the static strength of the container of the cleaning machine during operation and obtained the size and distribution of bearing stress of the container of the cleaning machine [12]. Kim set up a new guide frame and bogie according to the specification, and carried out stress analysis on them by using finite element method [13]. Although scholars have put forward the problems that may occur in the rail transit vehicle system, the safety of the vehicle system has not been quantitatively evaluated.

Aiming at the possible risk problems in each subsystem of rail transit vehicle system, scholars used a variety of methods to carry out risk assessment on rail transit vehicle system. In many current researches, analytic hierarchy process (AHP) is used to analyze the risk of rail transit vehicle system. AHP is a qualitative and quantitative evaluation method. It uses expert scoring rules to evaluate the importance of multiple decisions and form a judgment matrix, which can objectively reflect the actual situation [14]. Fan carried out a risk assessment for the complex maglev bogie system with multiple subsystem faults by using a combination of analytic hierarchy process and fuzzy comprehensive evaluation [15]. Ghodrati used analytic hierarchy process (AHP) and risk priority number to prioritize the faults of rail vehicles [16]. Li evaluated the security of urban rail transit power supply system by using analytic hierarchy process and comprehensive evaluation method [17].

However, there are inconsistency problems and low accuracy of calculation in the existing evaluation by AHP. The establishment of a judgment matrix in AHP originates from the scoring of the expert group, the obtained data is inevitably subjective, so the judgment matrix usually does not meet the consistency [18]. Meanwhile, the difficulty of safety evaluation of rail transit vehicle system lies in that there are many decision items in its index system. Carrying out comprehensive evaluation needs to take into account all aspects, and to compare the importance of various indexes [19]. Building an appropriate safety evaluation index system and evaluating the safety status is of great significance to improve the safety level of rail transit operation [20]. In view of this, based on the principle of AHP method, we studied a optimization model to correct the inconsistent judgment matrix, and used Genetic Algorithm to optimize the model. We call the improved AHP with GA as AHP-GA. Combined with fuzzy comprehensive evaluation method, we evaluate the safety of rail transit vehicle systems, to provide reference for the safe operation of rail transit company.

## Theoretical model

### Improved AHP algorithm

AHP is a concise and effective method to make decisions on complex problems [21]. With the development of science and technology, it is urgent to make quantitative research on the factors, things and concepts that can only be described qualitatively in the fields of society, economy, biology, psychology, organization, and management [22–24].

AHP combines qualitative analysis with quantitative analysis. According to the overall goal of the problem, it decomposes the problem into several factors from a systematic point of view and forms a hierarchical structure model according to its dominant relationship. Then it uses the method of pairwise comparison to determine the relative importance between decision schemes, so as to obtain satisfactory decision-making. The specific steps of AHP are as follows [25].

1. Establish hierarchical structure model;
2. Construct judgment matrix *A*;
$$A={\left(a_{\mathrm{ij}}\right)}_{n\times n}=(\begin{array}{cccc} a_{11} & a_{12} & \cdots & a_{1n}\\ a_{21} & a_{22} & \cdots & a_{2n}\\ \vdots & \vdots & & \vdots \\ a_{n1} & a_{n2} & \cdots & a_{nn} \end{array})$$ (1)
  where *a*_*ij*_ is the importance comparison result of the *i* factor with the *j* factor, *n* is the number of factors which need to be compared.
3. The traditional method for calculating the weight vector is the square root method (SRM), and the formula is as follows,
$$M=(m_{1},m_{2},\cdots ,m_{n}),m_{i}=\sqrt[{n}]{\prod_{i=1}^{n}a_{ij}}$$ (2)
$$W=(\omega_{1},\omega_{2},\cdots ,\omega_{n}),\omega_{i}=\frac{m_{i}}{\sum_{j=1}^{n}m_{j}},\;i,j=(1,2,\cdots ,n)$$ (3)
  where *m*_*i*_ is the weight value of each element. *ω*_*i*_ is the weight value of each element after normalization.
4. Consistency test of a judgment matrix. According to formula $\lambda \max=\frac{1}{n}\sum_{i=1}^{n}\frac{{\left(A\omega \right)}_{i}}{\omega_{i}}$, find the maximum characteristic root *λ*max, then calculate the formula $CR=\frac{CI}{RI},CI=\frac{\lambda_{\max}-n}{n-1}$, (*RI* is the mean-random consistency index obtained by some scholars with a large number of random matrices). Generally when *CR*<0.1, it is considered that the judgment matrix has satisfactory consistency.

From top to bottom, the hierarchical structure model of AHP are target layer *A*, primary decision-making layer *B*, and secondary decision-making layer *C*, etc. Layer *B* contains *n* decisions, namely *B*_1_, *B*_2_, …, *B*_*n*_. The second level decision-making layer contains *m* decisions, namely *C*_1_, *C*_2_, …, *C*_*m*_, so as to obtain the judgment matrix of layer $B={\left\{B_{ij},ij=1∼n\right\}}_{n\times n}$. The *C*-layer judgment matrix corresponding to the *B*-layer element *B*_*k*_. $C={\left[\overset{k}{C}{}_{ij},ij=1∼m;k=1∼n\right]}_{m\times n}$. The consistency test of the judgment matrix affects the iteration times and calculation accuracy of the weight calculation. At the same time, when the accuracy of the weight calculation results is not high, the judgment matrix should be modified [26].

The consistency test of the judgment matrix is the key step of AHP. According to the definition of judgment matrix, if the matrix completely meets the consistency, it is theoretically as follows,

$$\omega_{k}>0,\sum_{k=1}^{n}\omega_{k}=1,b_{ij}=\frac{\omega_{i}}{\omega_{j}}$$ (4)

where $k,i,j=1,2,\cdots ,n,b_{ii}=\frac{\omega_{i}}{\omega_{i}}=1,b_{ij}=\frac{1}{b_{ji}},b_{ik}=b_{kj}$.


$$\sum_{k=1}^{n}\left(b_{ik}\omega_{k}\right)=\sum_{k=1}^{n}\left(\frac{\omega_{i}}{\omega_{k}}\omega_{k}\right)=n\omega_{k},i=1∼n$$ (5)

$$\sum_{i=1}^{n}\left|\sum_{i=1}^{n}\left(b_{ik}\omega_{ik}\right)-n\omega_{i}\right|=0$$ (6)

As described above, the consistency problem of the judgment matrix is transformed into the nonlinear function optimization problem [27], and there is a eq,

$$\min\;CIF(n)=\frac{\sum_{i=1}^{n}\left|\sum_{i=1}^{n}\left(b_{ik}\omega_{ik}\right)-n\omega_{i}\right|}{n}$$ (7)

$$s.t.\;\omega_{k}>0,\sum_{k=1}^{n}\omega_{k}=1,k=1∼n$$

where *CIF*(*n*) is the consistency function of the judgment matrix. *ω* is the sorting weight of the element.

**Fig 2:**
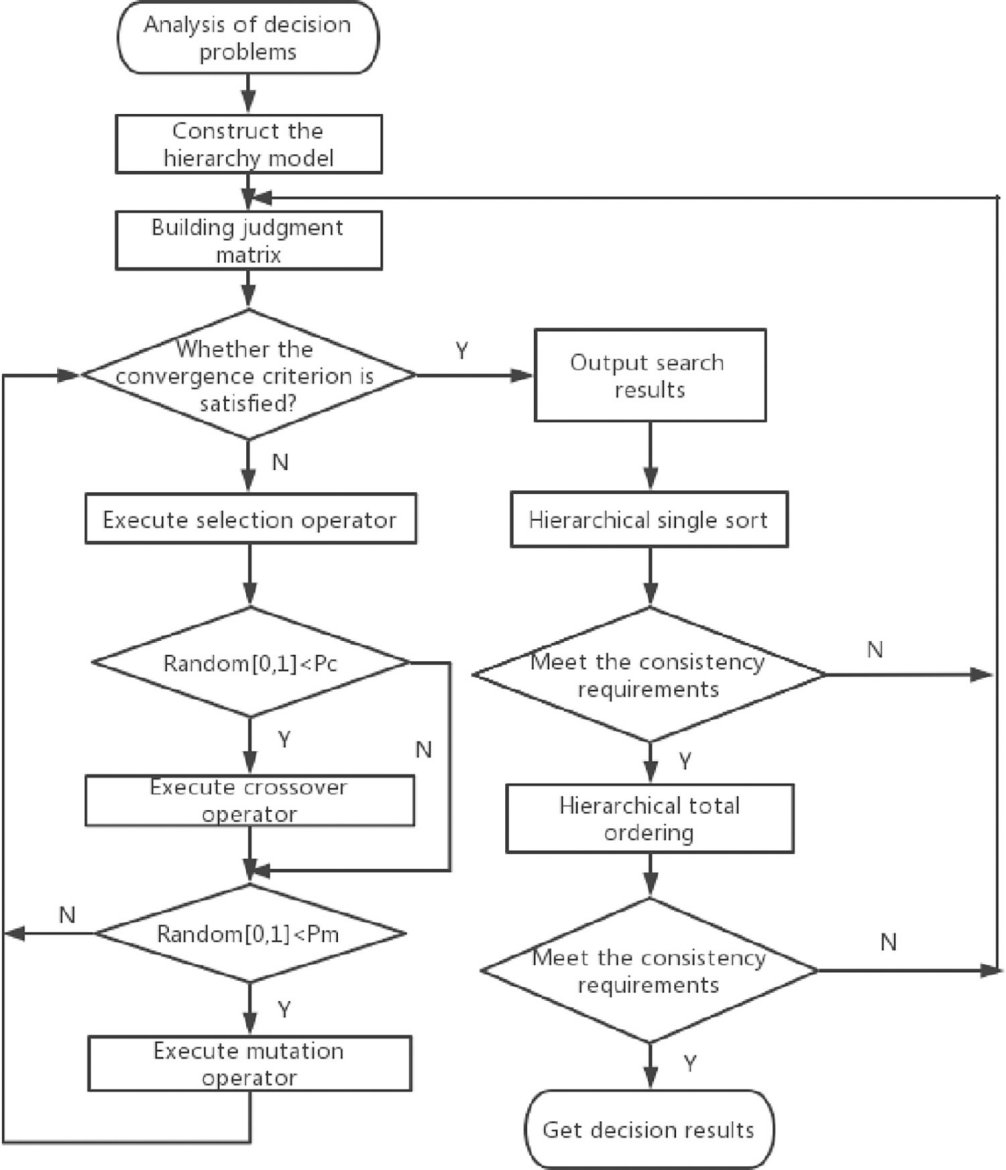
Optimized flow chart.

This function is difficult to deal with the analytic solution method. Genetic Algorithm (GA) is introduced to solve the function [28]. The overall search strategy and optimization search method of GA do not depend on gradient information or other auxiliary knowledge, but only need the objective function and corresponding fitness function that affect the search direction [29]. Therefore, genetic algorithm provides a general framework for solving complex system problems and is a classical function optimization method [30]. The main operations process of genetic algorithm include three basic operators: selection, crossover and mutation. The operation of individual genetic operators is carried out under the condition of random selection, so the migration rule of individual optimal solution in the population is random and efficient [31]. Fig 2 shows the AHP process with GA improvement.

### Fuzzy comprehensive evaluation

After operation, fuzzy comprehensive evaluation method was used to evaluate safety. Fuzzy comprehensive evaluation method is a comprehensive evaluation method based on fuzzy mathematics [32]. According to the membership theory of fuzzy mathematics, the comprehensive evaluation method transforms qualitative evaluation into quantitative evaluation, that is, fuzzy mathematics is used to make an overall evaluation of things or objects restricted by many factors [33]. It has the characteristics of clear results and strong systematicness. It can better solve the fuzzy and difficult to quantify problems and is suitable for the solution of various uncertain problems [34, 35]. Fuzzy comprehensive evaluation method is often used in fuzzy environment to make comprehensive decision for things affected by multiple factors. The model of fuzzy comprehensive evaluation is:

$$B=A\cdot R=(a_{1},a_{2},\cdots ,a_{m})(\begin{array}{cccc} r_{11} & r_{12} & \cdots & r_{1n}\\ r_{21} & r_{22} & \cdots & r_{2n}\\ \vdots & \vdots & & \vdots \\ r_{n1} & a_{n2} & \cdots & r_{nn} \end{array})=(b_{1},b_{2},\cdots ,b_{m})$$ (8)

where *b*_*j*_ (*j* = 1,2,…,*n*) is obtained by the *j* column operation of *A* and *R*, and represents the membership degree of the rated object to the fuzzy subset of *V*_*j*_ grade on the whole [36].

## Case analysis

The square root method and GA were used to solve the weights of the elements in the following two judgment matrices respectively. The results were showed in Table 1.


$$B_{1}=[\begin{array}{cccc} 1 & 3 & 6 & 5\\ 1/3 & 1 & 3 & 6\\ 1/6 & 1/3 & 1 & 2\\ 1/5 & 1/6 & 1/4 & 1 \end{array}]\;B_{2}=[\begin{array}{cccccc} 1 & 3 & 3 & 7 & 6 & 5\\ 1/3 & 1 & 1/3 & 5 & 5 & 3\\ 1/3 & 3 & 1 & 6 & 3 & 4\\ 1/7 & 1/5 & 1/6 & 1 & 1/3 & 1/4\\ 1/6 & 1/3 & 1/3 & 3 & 1 & 1/4\\ 1/5 & 1/3 & 1/4 & 4 & 4 & 1 \end{array}]$$

**Table 1:** The comparison of the weights.

Method	J-Matrix	Sorting weight of each element						*CIF*(*n*)
		*ω*_1_	*ω*_2_	*ω*_3_	*ω*_4_	*ω*_5_	*ω*_6_	
SRM	*B*_1_	0.5520	0.2690	0.1229	0.0562			0.1136
GA	*B*_1_	0.5714	0.699	0.1110	0.0478			0.0321
SRM	*B*_2_	0.4032	0.1674	0.2444	0.0310	0.0569	0.0971	0.1054
GA	*B*_2_	0.4263	0.1665	0.2546	0.0315	0.0422	0.0790	0.0433

It can be seen from the weight calculation results of the two judgment matrices in Table 1, that the weight obtained by the GA was more accurate than the square root method. The consistency test coefficient obtained by the square root method are 0.1136 and 0.1054, cannot make *B*_1_ and *B*_2_ pass the consistency test, because they are more than 0.1. While the result obtained by GA are 0.0321 and 0.0433, can make *B*_1_ and *B*_2_ pass the consistency test.

Weight calculation and matrix correction were performed simultaneously when GA is applied. The following matrices *B*_1_* and *B*_2_* are the final correction matrices of *B*_1_ and *B*_2_. We can see that the consistency of matrices *B*_1_* and *B*_2_* is significantly better than that of matrices *B*_1_ and *B*_2_.


$$B_{1}*=[\begin{array}{cccc} 1 & 0.8175 & 5.1477 & 11.9540\\ 1.2233 & 1 & 6.2973 & 14.6234\\ 0.1943 & 0.1588 & 1 & 2.3222\\ 0.0837 & 0.0684 & 0.4306 & 1 \end{array}]\;B_{2}*=[\begin{array}{cccccc} 1 & 2.5604 & 1.6744 & 13.53333 & 10.1019 & 5.6092\\ 0.3906 & 1 & 0.6540 & 5.2857 & 3.9455 & 2.1908\\ 0.5972 & 1.5291 & 1 & 8.0825 & 6.0332 & 3.2500\\ 0.0739 & 0.1892 & 0.1237 & 1 & 0.7464 & 0.4145\\ 0.0990 & 0.2535 & 0.1658 & 1.3397 & 1 & 0.5553\\ 0.1783 & 0.4565 & 0.2985 & 2.4127 & 1.8009 & 1 \end{array}]$$

## Fuzzy comprehensive evaluation example

### Establishment of safety evaluation index system for rail transit vehicle system

**Fig 3:**
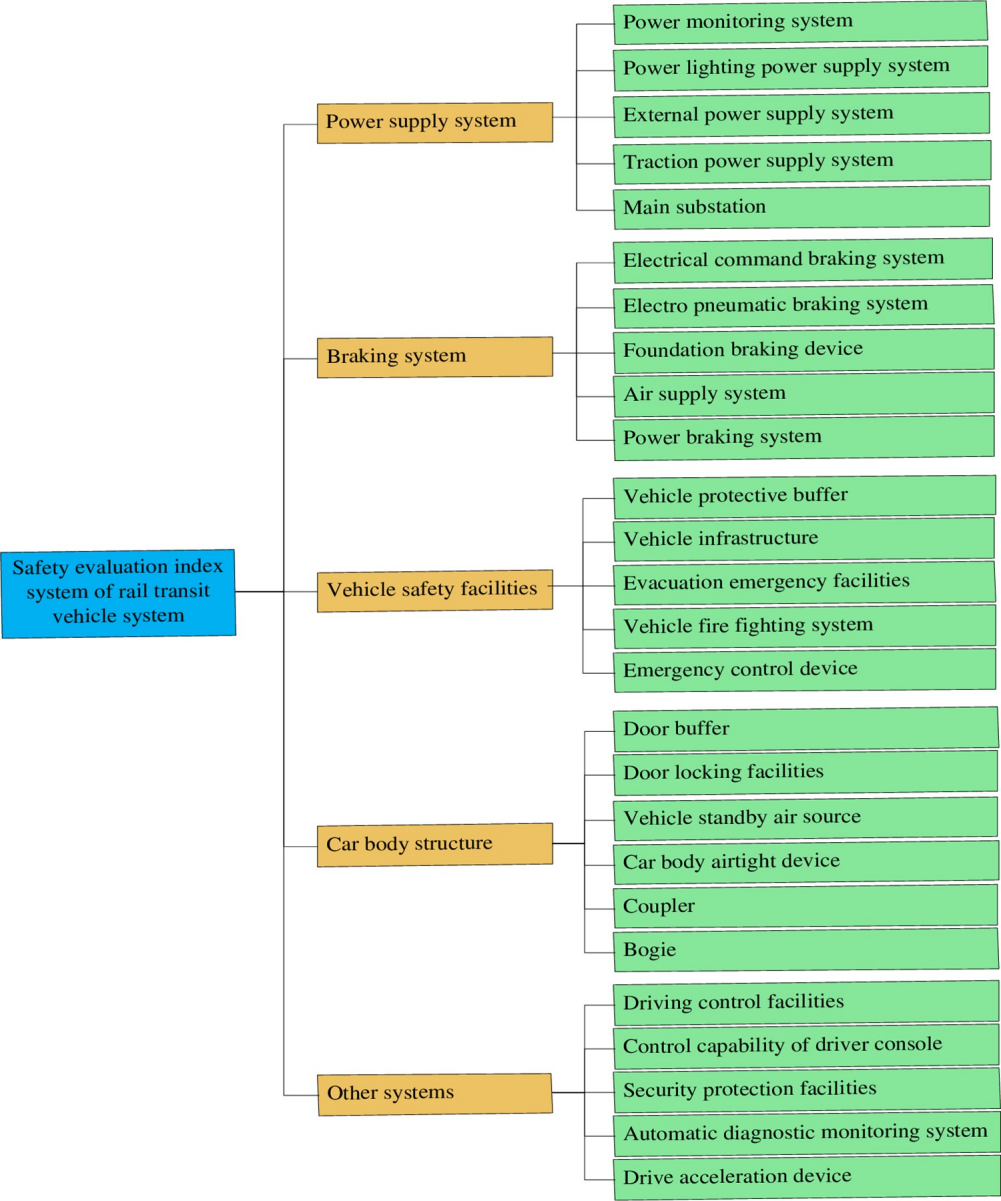
Safety evaluation index system of rail vehicle system.

According to the *SMART* principle, the safety evaluation index system of rail transit vehicle system is constructed. *SMART* was originally the first letter combination of five English words. Combined with the operating characteristics of rail transit equipment, *SMART* principle is understood as the combination of words such as "significant", "measurable", "attachable", "relevant", and "tangible", that is, five principles such as significance, operability, practicability, relevance, and specificity [37]. Based on this, the overall objective of rail transit vehicle system index selection is divided into several operable objectives. Statistical analysis of rail transit vehicle system accidents, combined with relevant standards, specifications, and relevant documents of vehicle system [7, 38, 39]. Organize experts to hold a meeting by brainstorming method to summarize and select the main factors of vehicle system indicators. According to Likert’s five-level scale method, the index system is improved by using the method of network questionnaire [40], a questionnaire survey was conducted on the rationality of the selection of index factors and the importance of indicators. A total of 493 questionnaires were distributed and 471 valid questionnaires were recovered, included 432 rail transit operation management and staff, 22 rail transit design and research personnel, and 17 teachings and research personnel in the direction of rail transit safety management in colleges and universities. The Cronbach’s alpha reliability coefficient of the questionnaire was greater than 0.8, the KMO value was 0.885, and the p-value of Bartlett’s ball test was less than 0.001, indicated that the formulation of the questionnaire was reasonable. Through comprehensive analysis of relevant standards, expert opinions, and research results, the safety evaluation index system of rail transit vehicle system was obtained, as showed in Fig 3.

### Factor set and comment set

As shown in Fig 3, the top layer is target layer *A*, *Safety evaluation index system of rail transit vehicle system*. And the middle layer is decision layer I, *A* = {*B*_1_, *B*_2_, *B*_3_, *B*_4_, *B*_5_}, *B*_1_ is *Power supply system*, *B*_2_ is *Braking system*, *B*_3_ is *Vehicle safety facilities*, *B*_4_ is *Car body structure*, and *B*_5_ is *Other systems*. *B* is decision layer II, composed of *C*. *B*_1_ = {*C*_11_, *C*_12_, *C*_13_, *C*_14_, *C*_15_}, *B*_2_ = {*C*_21_, *C*_22_, *C*_23_, *C*_24_, *C*_25_}, *B*_3_ = {*C*_31_, *C*_32_, *C*_33_, *C*_34_, *C*_35_}, *B*_4_ = {*C*_41_, *C*_42_, *C*_43_, *C*_44_, *C*_45_, *C*_46_}, *B*_5_ = {*C*_51_, *C*_52_, *C*_53_, *C*_54_, *C*_55_}. For *A*, *B*, and *C*, the comment set has 5 levels, *V* = {very poor, poor, average, good, very good}.

### Establishment of judgment matrix

Through the comparison of importance, the judgment matrices of the decision-making level were as follows,

$$A=[\begin{array}{ccccc} 1 & 3 & 3 & 2 & 4\\ 1/3 & 1 & 1 & 1/2 & 2\\ 1/3 & 1 & 1 & 1/2 & 2\\ 1/2 & 2 & 2 & 1 & 2\\ 1/4 & 1/2 & 1/2 & 1/2 & 1 \end{array}[,\;B_{1}=[\begin{array}{ccccc} 1 & 5 & 3 & 3 & 2\\ 1/5 & 1 & 1/2 & 1/2 & 1/2\\ 1/3 & 1 & 1 & 1 & 1/2\\ 1/3 & 2 & 2 & 1 & 1/3\\ 1/2 & 2 & 2 & 3 & 1 \end{array}]$$

$$B_{2}=[\begin{array}{ccccc} 1 & 4 & 2 & 3 & 4\\ 1/4 & 1 & 1/2 & 1/2 & 2\\ 1/2 & 2 & 1 & 1 & 2\\ 1/3 & 2 & 1 & 1 & 2\\ 1/4 & 1/2 & 1/2 & 1/2 & 1 \end{array}],\;B_{3}=[\begin{array}{ccccc} 1 & 3 & 4 & 2 & 4\\ 1/3 & 1 & 1 & 1/2 & 2\\ 1/4 & 1 & 1 & 1/2 & 3\\ 1/2 & 2 & 2 & 1 & 2\\ 1/4 & 1/2 & 1/3 & 1/2 & 1 \end{array}]$$

$$B_{4}=[\begin{array}{cccccc} 1 & 4 & 2 & 7 & 5 & 5\\ 1/4 & 1 & 1/2 & 2 & 2 & 2\\ 1/2 & 2 & 1 & 3 & 2 & 2\\ 1/7 & 1/2 & 1/3 & 1 & 1/2 & 1/2\\ 1/5 & 1/2 & 1/2 & 2 & 1 & 1\\ 1/5 & 1/2 & 1/2 & 2 & 1 & 1 \end{array}],\;B_{5}=[\begin{array}{ccccc} 1 & 2 & 4 & 2 & 4\\ 1/2 & 1 & 2 & 1 & 3\\ 1/4 & 1/2 & 1 & 1/2 & 1\\ 1/2 & 2 & 2 & 1 & 3\\ 1/4 & 1/3 & 1 & 1/3 & 1 \end{array}]$$

### Calculation results of weight and consistency function

Although the above judgment matrices are determined after repeated comparative analysis, they are still not completely consistent. The weight of each element is calculated by GA and square root method (SRM), and the results are shown in Table 2. As can be seen from the table, *CIF* values are all less than 0.1, indicating that these judgment matrices can pass the consistency test. However, by contrast, the GA method proposed in this paper has a better consistency level because of the lower *CIF* values. This shows that the improved AHP using genetic algorithm can calculate more accurate results compared with SRM method.

**Table 2:** Calculations of the weight.

J-Matrix	*ω*_1_	*ω*_2_	*ω*_3_	*ω*_4_	*ω*_5_	*ω*_6_	*CIF*	*CIF*
							GA	SRM
*A*	0.4076	0.1392	0.1381	0.2315	0.0838		0.0121	0.0131
*B*_1_	0.4110	0.0660	0.1195	0.1482	0.2553		0.0143	0.0389
*B*_2_	0.4283	0.1137	0.1960	0.1817	0.0804		0.0115	0.0175
*B*_3_	0.4270	0.1309	0.1417	0.2266	0.0739		0.0174	0.0309
*B*_4_	0.4370	0.1338	0.1993	0.0508	0.0881	0.0910	0.0129	0.0136
*B*_5_	0.3854	0.2083	0.0778	0.2501	0.0784		0.0468	0.0488

### Determination of membership

The membership degree describes the membership degree of an element to its fuzzy subset in the fuzzy comprehensive evaluation method. The membership matrix of the decision-making level is obtained by expert scoring and membership function *R* = {*R*_1_, *R*_2_, *R*_3_, *R*_4_, *R*_5_}. Ten experts were invited to vote for each element of the second level of decision-making, and the evaluation matrix is as follows,

$$R_{1}=[\begin{array}{ccccc} 0 & 0.1 & 0.4 & 0.3 & 0.2\\ 0 & 0.2 & 0.3 & 0.5 & 0\\ 0 & 0.3 & 0.2 & 0.4 & 0.1\\ 0 & 0.2 & 0.5 & 0.2 & 0.1\\ 0 & 0 & 0.5 & 0.4 & 0.1 \end{array}]\;R_{2}=[\begin{array}{ccccc} 0 & 0.1 & 0.4 & 0.4 & 0.1\\ 0 & 0.1 & 0.4 & 0.5 & 0\\ 0 & 0.2 & 0.3 & 0.4 & 0.1\\ 0 & 0.2 & 0.5 & 0.2 & 0.1\\ 0 & 0.1 & 0.4 & 0.5 & 0 \end{array}]$$

$$R_{3}=[\begin{array}{ccccc} 0 & 0.1 & 0.5 & 0.4 & 0\\ 0 & 0.1 & 0.4 & 0.5 & 0\\ 0 & 0.1 & 0.4 & 0.4 & 0.1\\ 0 & 0.2 & 0.5 & 0.3 & 0\\ 0 & 0 & 0.5 & 0.5 & 0 \end{array}]\;R_{4}=[\begin{array}{ccccc} 0 & 0.1 & 0.4 & 0.3 & 0.2\\ 0 & 0.2 & 0.3 & 0.5 & 0\\ 0 & 0.2 & 0.3 & 0.4 & 0.1\\ 0 & 0.2 & 0.5 & 0.2 & 0.1\\ 0 & 0.1 & 0.4 & 0.3 & 0.2\\ 0.1 & 0.2 & 0.4 & 0.3 & 0 \end{array}]$$

$$R_{5}=[\begin{array}{ccccc} 0 & 0.1 & 0.4 & 0.4 & 0.1\\ 0 & 0.1 & 0.4 & 0.5 & 0\\ 0 & 0.2 & 0.3 & 0.4 & 0.1\\ 0 & 0.2 & 0.5 & 0.3 & 0\\ 0 & 0.2 & 0.3 & 0.4 & 0.1 \end{array}]$$

### Scoring results

Scored results *D* = *ω*_*A*_·*C*, *ω*_*A*_, it is the weight of the elements of the decision layer, *C* = {*c*_1_; *c*_2_; *c*_3_; *c*_4_; *c*_5_}, *c*_*i*_ = *ω*_*i*_·*R*_*i*_, *i* = 1, 2, 3, 4, 5. Finally, the evaluation grade was determined accorded to the principle of maximum membership. The calculation results were follows,

$$C=[\begin{array}{ccccc} 0 & 0.1198 & 0.4099 & 0.3359 & 0.1345\\ 0 & 0.1378 & 0.3986 & 0.3831 & 0.0806\\ 0 & 0.1153 & 0.4728 & 0.3979 & 0.0142\\ 0.0091 & 0.1475 & 0.3718 & 0.3416 & 0.1300\\ 0 & 0.1406 & 0.4094 & 0.3958 & 0.0542 \end{array}]$$

D=[0.00210.12990.40820.35740.1026]

According to the above *V* = {very poor, poor, average, good, very good}, the comment set has 5 levels. Based on the principle of maximum membership, we can get the evaluation result. *D* = [0.0021 0.1299 0.4082 0.3574 0.1026], the maximum element is 0.4082, and the comment level of the element position of 0.4082 is average. So the evaluation result of the target layer is "average".

## Conclusions

1. Analytic hierarchy process (AHP) is widely used in safety and environment assessment. The consistent binary contrast matrix is the key to the successful application of AHP. However, for high-order binary contrast matrices, an inconsistency usually exists. Based on the principle of the binary contrast matrix, the genetic algorithm is applied to correct the inconsistent binary contrast and to calculate each element’s weight of the binary contrast matrix, which can solve the inconsistency of high-order binary contrast matrices.
2. A safety evaluation index system for the rail transit vehicle system is constructed. The system is a hierarchical organization, including 5 second-level indicators and 26 three-level indicators. A comprehensive safety evaluation of the rail transit vehicle system is conducted. The modified hierarchical analysis method based on a genetic algorithm is applied to calculate the system index weight to make the evaluation results more accurate.
3. Based on the constructed rail transit safety evaluation system and the comprehensive safety evaluation method, the vehicle system safety evaluation of a rail transit enterprise is applied. The evaluation result level is "average", indicating that the comprehensive safety level of the enterprise needs to be improved.

## Supporting information

S1 File (ZIP)

S1 Data (XLSX)
